# Evidence for a LOS and a capsular polysaccharide in *Capnocytophaga canimorsus*

**DOI:** 10.1038/srep38914

**Published:** 2016-12-15

**Authors:** Francesco Renzi, Simon J. Ittig, Irina Sadovskaya, Estelle Hess, Frederic Lauber, Melanie Dol, Hwain Shin, Manuela Mally, Chantal Fiechter, Ursula Sauder, Mohamed Chami, Guy R. Cornelis

**Affiliations:** 1 Université de Namur, URBM, 5000 Namur, Belgium; 2 Infection Biology, Biozentrum der Universität Basel, CH4056 Basel, Switzerland; 3 Université du Littoral-Côte d’Opale, Institut Charles Violette, EA7394, 62327 Boulogne-sur-mer, France; 4 BioEM lab, Biozentrum der Universität Basel, CH4056 Basel, Switzerland

## Abstract

*Capnocytophaga canimorsus* is a dog’s and cat’s oral commensal which can cause fatal human infections upon bites or scratches. Infections mainly start with flu-like symptoms but can rapidly evolve in fatal septicaemia with a mortality as high as 40%. Here we present the discovery of a polysaccharide capsule (CPS) at the surface of *C. canimorsus* 5 (Cc5), a strain isolated from a fulminant septicaemia. We provide genetic and chemical data showing that this capsule is related to the lipooligosaccharide (LOS) and probably composed of the same polysaccharide units. A CPS was also found in nine out of nine other strains of *C. canimorsus*. In addition, the genomes of three of these strains, sequenced previously, contain genes similar to those encoding CPS biosynthesis in Cc5. Thus, the presence of a CPS is likely to be a common property of *C. canimorsus*. The CPS and not the LOS confers protection against the bactericidal effect of human serum and phagocytosis by macrophages. An antiserum raised against the capsule increased the killing of *C. canimorsus* by human serum thus showing that anti-capsule antibodies have a protective role. These findings provide a new major element in the understanding of the pathogenesis of *C. canimorsus*.

*Capnocytophaga canimorsus* (formerly Centers for Disease Control group DF-2) are capnophilic Gram-negative bacteria that belong to the family of Flavobacteriaceae in the phylum Bacteroidetes. *C. canimorsus* is found in the normal oral flora of dogs and cats. Since its discovery in 1976, it is regularly isolated from severe human infections following contact with a dog or a cat^1^,^2^,^3^. The incidence of the infection has been estimated to 0.5 and 0.63 case/million inhabitants per year in Denmark and the Netherlands respectively^4^,^5^. However, a recent study carried out in Helsinki concluded that the incidence was of 4.1 cases/million inhabitants per year^6^ thus showing that the disease is more frequent than previously thought and that it is underestimated probably because of under-diagnosis mainly due to the fastidious growth of these bacteria in culture. *C. canimorsus* infections generally begin with flu symptoms and evolve in a few days into fulminant septicaemia and peripheral gangrene with mortality as high as 40%^1^,^4^,^7^,^8^,^9^. Splenectomy, alcohol abuse and immunosuppression have been associated with a number of cases, but more than 40% of the patients have no obvious risk factor^10^,^11^. Recent observations help understanding the infectiveness of *C. canimorsus* for humans. *C. canimorsus* manifest some resistance to phagocytosis by human polymorphonuclear leukocytes and detection by macrophages^12^,^13^, which results in a lack of release of pro-inflammatory cytokines^14^. Like many Gram-negative pathogens, *C. canimorsus* resist the bactericidal activity of 10% human or rabbit serum^13^,^15^ but they are nevertheless killed by undiluted fresh serum or blood^16^,^17^. In addition to the passive evasion from innate immunity, some strains are able to block the killing of *E. coli* phagocytosed by macrophages^12^,^18^ and to block the onset of pro-inflammatory signaling induced by an *E. coli* LPS stimulus^14^. *C. canimorsus* also have the unusual property to deglycosylate mammalian proteins, including IgG, IgM and surface glycoproteins from phagocytes^19^,^20^,^21^,^22^.

Gram-negative bacteria have a complex set of surface polysaccharides, which contribute to pathogenicity as well as commensalism^23^,^24^,^25^,^26^,^27^,^28^,^29^,^30^,^31^,^32^. These include the lipopolysaccharide (LPS) as well as capsular polysaccharides (CPS) or exopolysaccharides (EPS)^33^. The LPS, a major component of the outer membrane and one of the most pro-inflammatory bacterial compounds, consists of three regions: lipid A, which is generally responsible for triggering inflammation^34^,^35^, the core oligosaccharide, and the O-antigen. The *C. canimorsus* LPS is 100 fold less endotoxic than the highly immunogenic *E. coli* LPS^14^,^36^. Surprisingly, the lipid A alone, which is penta-acylated, lacking the 4′ phosphate and harboring a 1 phosphoethanolamine (P-Etn) at 2-amino-2-deoxy-D-glucose (GlcN) is almost not pro-inflammatory at all and thus the low endotoxic activity observed is conferred by the core oligosaccharide^36^. The LPS O-antigen of Gram-negative bacteria greatly varies between and within species, providing the main basis for serotyping. It can be a virulence factor contributing to serum resistance^27^,^28^,^30^,^31^,^32^,^37^,^38^,^39^,^40^,^41^,^42^ and O-antigen deficient strains of different bacteria have generally reduced virulence^30^,^31^. The O-antigen is synthesized independently of the lipid A-core^35^ and generally consists of several repeats of an identical oligosaccharide called the O-unit. Three pathways have been described for LPS biosynthesis and translocation, which essentially differ by their export mechanism. According to the main proteins involved, they are called Wzy-dependent, ABC-transporter dependent and synthase dependent^43^. Outside the LPS, many Gram-negative bacteria, including *E. coli*, have a capsular structure that increases their resistance towards the innate immune system (for review see^44^). These structures have various compositions and are assembled by diverse pathways. In *E. coli*, on the basis of genetics and biochemical criteria, capsules have been divided in four main groups. The biosynthesis and assembly mechanisms of capsules are closely related to those of the LPS and so group I and IV capsules are assembled by the Wzy-dependent pathway while group II and III capsules are assembled by ABC transporter-dependent pathways (for review see^33^). In the Wzy-dependent pathway the LPS O-chain is composed of repeating O-units that are polymerized by the activity of the Wzy polymerase^43^. The large structural diversity between O-antigens is the result of variations in the composition, sequence, and linkage of the O-units. The O-antigen repeating units are first assembled in the cytoplasm, linked to the lipid carrier undecaprenol phosphate (und-PP) and then transported across the inner membrane to the periplasm by the Wzx flippase. The und-PP-linked O-units are then polymerized on the periplasmic leaflet of the inner membrane by the Wzy polymerase that allows the formation of the complete O-chain. The O-chain is finally ligated to the core oligosaccharide by the WaaL ligase forming a complete LPS that is then translocated to the bacterial surface by the Lpt-system^45^. In group I and IV capsules, the O-units, assembled by the Wzx/Wzy-dependent pathway, are translocated to the bacterial surface by the coordinated action of the Wza, Wzb and Wzc proteins^46^. The Wza proteins form a channel in the outer membrane allowing capsule export and the translocation is controlled by the coordinated activity of the Wzc tyrosine autokinase and its cognate phosphatase Wzb^47^,^48^.

The *C. canimorsus* lipid A and core oligosaccharide chemical structures were recently established^36^,^49^. To date, no data are available on the composition and structure of the *C. canimorsus* LPS O-antigen.

Here we show that *C. canimorsus* 5 possesses a lipooligosaccharide (LOS) rather than a LPS and a capsular polysaccharide (CPS) likely made of the same O-antigen repeating units. The CPS and not the LOS increases resistance to human serum, polymyxin B and phagocytosis by macrophages. Finally, antibodies raised against the LOS O-antigen and CPS have a protective role.

## Results

### *C. canimorsus* 5 has a high molecular weight polysaccharide related to the O-antigen

**Figure 1:**
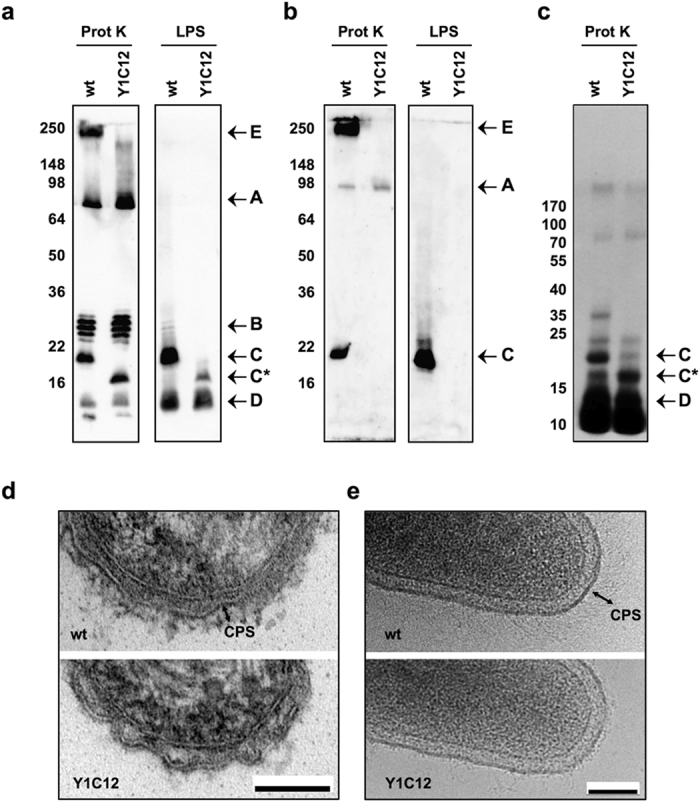
Y1C12 mutant shows an altered LPS and lacks a putative CPS. (**a**) Immunoblot analysis of proteinase K-treated wt Cc5 and Y1C12 mutant bacterial lysates and of LPS isolated from wt Cc5 and Y1C12 mutant using anti-Cc5 serum. (**b**) Immunoblot analysis as described in (**a**) using Y1C12-adsorbed anti-Cc5 serum. (**c**) Proteinase K-resistant structures of wt Cc5 and Y1C12 mutant that had been labeled *in vivo* using [9,10-^3^H] palmitic acid. Electron micrographs of thin sections (**d**) and frozen-hydrated (**e**) wt and Y1C12 bacteria. The double arrow indicates the capsule (CPS). The scale bar is 100 nm.

In our previous work we isolated a *C. canimorsus* 5 (Cc5) transposon mutant, named Y1C12, which was hypersensitive to killing by human complement and phagocytosis^13^. The mutation inactivated a putative glycosyltransferase gene suggesting that the Y1C12 mutant was affected at the level of a polysaccharide structure, likely the LPS O-antigen^13^,^49^. Analysis of bacterial polysaccharide structures by digesting whole bacteria with proteinase K and detection by western blot using both a crude anti-Cc5 serum and a Y1C12-adsorbed anti-Cc5 serum showed that *C. canimorsus* 5 has several polysaccharide structures (Fig. 1a,b and ref. 13). Two of them, a low molecular weight structure (band C in Fig. 1 and ref. 13) and a high molecular weight one (band E in Fig. 1) appeared to be affected in the Y1C12 mutant. While the low molecular weight band was shifted in size (band C* in Fig. 1a and ref. 13), the high molecular weight structure was completely missing in the Y1C12 mutant strain (Fig. 1a and b). Since the low molecular weight structure (band C), and its smaller version (band C*), could be isolated by LPS purification procedures with phenol-chloroform-petroleum ether^36^ (Fig. 1a) and labeled by tritiated palmitate (Fig. 1c and ref. 13), we conclude that it represents the LPS. However, the absence of a ladder-like pattern suggested that it is composed of a limited number of O-antigen repeat units and thus represents a lipooligosaccharide (LOS)^50^ rather than a LPS. Another structure, band D in Fig. 1a, could also be isolated by LPS purification procedures^36^ (Fig. 1a) and was labeled by tritiated palmitate (Fig. 1c) but was not affected in the Y1C12 mutant suggesting that it could represent an additional LOS/LPS form different from band C. In contrast, the high molecular weight structure, present in the wt strain but missing in the Y1C12 mutant (band E), could not be isolated by LPS purification methods (Fig. 1a) and was not labeled by tritiated palmitate (Fig. 1c and ref. 13) suggesting that it could represent a capsular polysaccharide (CPS) not linked to the lipid A-core. The absence of this putative capsule in the Y1C12 strain together with the presence of an altered LOS, suggested that the biosynthetic pathways of the CPS and the O-antigen could be partially common. This view is reinforced by the fact that both the putative CPS (band E) and the wt LOS (band C) were recognized by the Y1C12-adsorbed antiserum (Fig. 1b). Assuming the epitope recognized by the Y1C12-adsorbed antiserum is the O-antigen repeating unit (or parts thereof), one could infer that band C corresponds to LOS and that the chemical structure of the repeating unit (or parts thereof) of the CPS and LOS O-antigen could be similar if not identical. The gene inactivated in mutant Y1C12 turned out to be *Ccan_23370*^51^, now annotated as *wbuB*, an *N-*acetylfucosamine (FucNAc) transferase, suggesting that FucNAc could be part of both the LOS O-antigen and the CPS.

### A capsule is present at the bacterial surface

In order to visualize the *C. canimorsus* CPS at the bacterial surface we analyzed thin sections of resin embedded wt and Y1C12 mutant *C. canimorsus* bacteria by transmission electron microscopy. As shown in Fig. 1d, a thick layer was present at the bacterial surface adjacent to the outer membrane of wt cells while it was missing in the Y1C12 strain. This layer was also visualized by cryo-electron microscopy on frozen-hydrated wt bacteria (Fig. 1e). This technique, preserving the structures integrity, allowed estimating the layer thickness to around 50 nm. This layer thus represents the Cc5 CPS and resembles the capsular polysaccharides described in *Bacteroides fragilis*^52^.

Overall, these data indicate that *C. canimorsus* 5 possesses a high molecular weight polysaccharidic structure related to the LOS O-antigen. This structure could thus be a group I or IV capsule made out of O-antigen repeating units^33^.

### Identification of the genes encoding the capsule and the O-antigen of *C. canimorsus* 5

**Figure 2:**
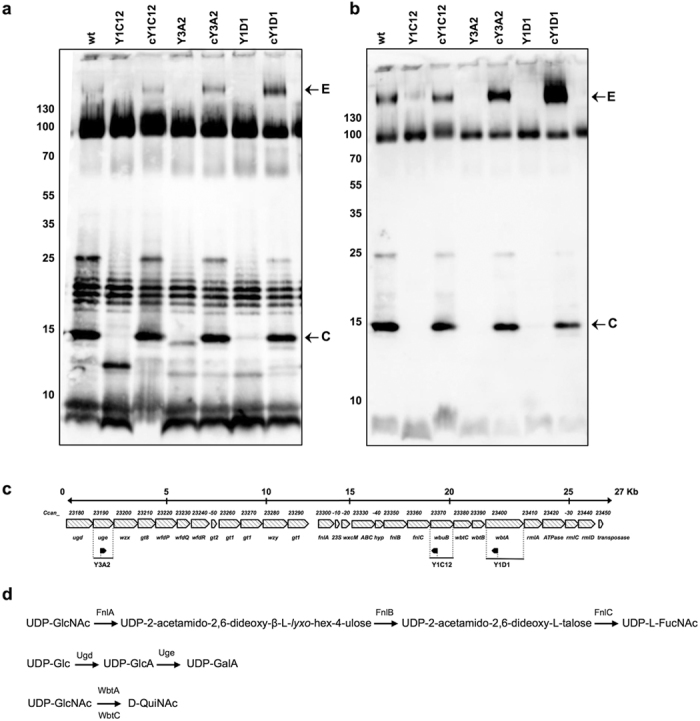
Identification of the Cc5 O-antigen cluster. (**a**) Immunoblot analysis of proteinase K-treated wt Cc5 and Tn4351 mutated Cc5 using anti-Cc5 serum. (**b**) Immunoblot analysis as described for panel (**a**) using Y1C12-adsorbed anti-Cc5 serum. (**c**) Genetic organization of the O-antigen cluster of Cc5. Extents, orientation and names of the genes are indicated. Flags indicate position and orientation of Transposon 4531 insertions. (**d**) Representation of the sugar biosynthetic pathways affected in the transposon mutants. Adapted from^53^.

In order to see whether CPS and O-antigen would share the same biosynthetic pathways, we first identified the genes involved in the biosynthesis of the capsule and the LOS O-antigen monosaccharides. To this aim we took advantage of the transposon Tn4351 library that previously allowed us to identify the Y1C12 mutant^13^. We isolated two other mutants (Y1D1 and Y3A2) whose survival rate in 10% human serum was severely decreased compared to that of the wt *C. canimorsus* 5 (Supplementary Fig. S1). Analysis of bacterial polysaccharide structures of Y1D1 and Y3A2 by western blot using both a crude anti-Cc5 serum and a Y1C12-adsorbed anti-Cc5 serum showed that both mutants, like Y1C12, lacked the capsular polysaccharide as well as the wt LOS band (band C and E in Fig. 2a and b). The integration sites of the transposon in Y1D1 and Y3A2 were mapped on the *C. canimorsus* 5 genome sequence^51^ and were found to be located in different genes clustered with *wbuB* (Y1C12)^13^ in one locus (Fig. 2c). This locus encodes 28 genes, whereof 23 are predicted to be involved in sugar synthesis, transfer or export. In Y1D1 the transposon inserted in gene *wbtA* (*wbtA* in *F. tularensis* or *wbpM* in *P. aeruginosa*), a gene required for *N*-acetylquinovosamine (QuiNAc) biosynthesis^53^,^54^,^55^ (Fig. 2c and d). For Y3A2, the transposon was found in gene *uge*, whose product converts D-glucuronate (GlcA) into D-galacturonate (GalA) (Fig. 2c and d). These data thus suggest that FucNAc, QuiNAc and GalA or derivatives thereof are part of the *C. canimorsus* 5 CPS and LOS-O-antigen.

### The capsule assembly is Wzx/Wzy dependent

To further clarify the relation between the capsule and LOS, we investigated their transport and assembly pathway. In the gene cluster previously identified (Fig. 2c) *Ccan_23200* shows sequence similarity to Wzx, the O-antigen flippase involved in the transport of O-antigen units to the periplasm across the inner membrane^43^,^56^ while *Ccan_23280* has sequence similarity to the O-antigen polymerase Wzy involved in the polymerization of the O-antigen repeating units, thus suggesting that the *C. canimorsus* 5 O-antigen and/or the CPS are synthesized by a Wzx/Wzy dependent pathway^43^. In order to verify this, we generated a *Ccan_23200* (putative *wzx*) mutant strain and analyzed its LOS and CPS by western blot. Mutation of *wzx* turned out to be polar but could be complemented by *wzx* plus *Ccan_23210* (Supplementary Data S1 and Supplementary Fig. S2). Even when properly complemented, the *wzx* mutant was still unable to produce the LOS and the CPS suggesting that *Ccan_23200* could indeed encode for the Wzx O-chain transporter (Supplementary Data 1 and Supplementary Fig. S2).

Deletion of the putative polymerase *wzy* gene (*Ccan_23280*) determined the disappearance of the CPS, significantly reduced normal LOS biosynthesis and led to the formation of low molecular weight LOS bands (Supplementary Fig. S2). However, the *wzy* mutation turned out to be polar on *Ccan_23290* encoding a glycosyltransferase (Supplementary Data S2 and Supplementary Fig. S2). Expression of *Ccan_23290* in the *wzy* mutant restored the wt LOS profile but not the CPS (Supplementary Fig. S2), suggesting that *Ccan_23280* indeed encodes the Wzy O-antigen polymerase that is responsible for the assembly of the CPS but not of the LOS O-chain. This also indicates that LOS band C represents the lipid A-core plus only one O-antigen unit. Notably, a second LOS band (band C^#^), with a higher molecular weight than band C, can be observed in the wt strain (Supplementary Fig. S2). This band was absent in the *wzy* (*Ccan_23290*^+^) strain thus indicating that it could represent a LOS with more than one O-antigen unit. Co-expression of *wzy* and *Ccan_23290* in the *wzy* mutant strain not only restored the CPS and LOS (band C and C^#^) profiles but also determined the formation of an LPS-like ladder pattern (Supplementary Fig. S2) suggesting that over-expression of *wzy* determines the formation of O-chains of different length. This pattern allows to deduce that band C^#^ consists of five repeated units. All together these data indicate that Cc5 possesses: a low molecular weight LOS (band C) composed of one O-antigen unit, a higher molecular weight LOS (band C^#^) likely composed of five O-antigen repeats and a capsular polysaccharide made of O-antigen units polymerized by Wzy.

### The capsular polysaccharide and the O-antigen repeating units share a structural similarity

**Figure 3:**
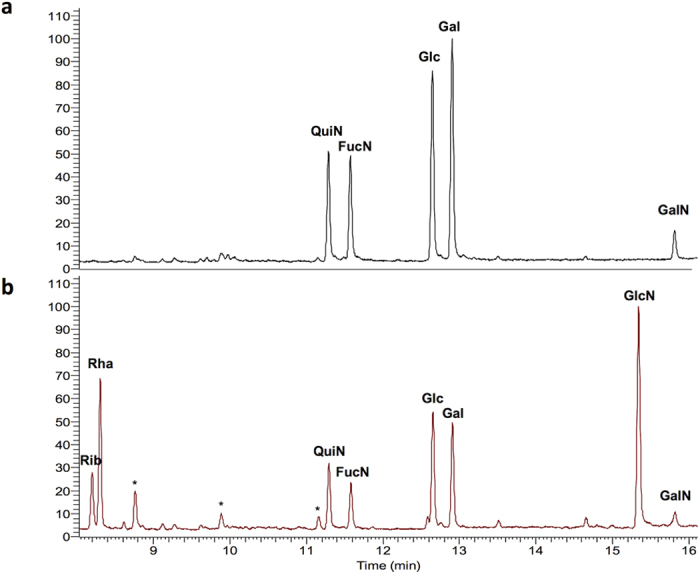
Cc5 O-antigen and capsule monosaccharide composition. GC-MS monosaccharide profiles of the CPS (**a**) and LOS O-antigen (**b**) preparations of wt Cc5. *non-sugar impurities.

In order to further corroborate that Cc5 CPS and the LOS O-antigen share common elements, we attempted to isolate both Cc5 LOS O-antigen and CPS and to determine their chemical composition. To this aim, Cc5 bacteria were subjected to hot phenol extraction^57^. Preliminary experiments identified carbohydrate molecules in both aqueous and phenol phases, therefore phenol extracts were further processed without phase separation. Both crude extracts were treated with hot dilute AcOH and fractionated on a Sepharose S-300 gel filtration chromatography column. Two major carbohydrate fractions, a high molecular weight (HMW) and a low molecular weight (LMW), were collected, in agreement with the electrophoretic profiles observed by western blot analysis. The HMW fraction eluted close to the void volume of the column (MW~200–300 kDa), and was more abundant in the aqueous phase preparation. Western blot analysis of the phenol extract showed a profile identical to that of the proteinase K digested bacteria, the aqueous phase being enriched with the HMW band E, the CPS. GC-MS analysis of this fraction showed that it contained QuiN, FucN, glucose (Glc), galactose (Gal) and galactosamine (GalN) (Fig. 3a). In addition, a colorimetric assay^58^ showed the presence of uronic acid.

The LMW fraction, presumably corresponding to the carbohydrate part of LOS bands C, C^#^ and D, was further fractionated on a BioGel P2 column, showing a heterogeneous profile. The major carbohydrate-positive fraction contained QuiN, FucN, Glc, Gal and GalN (Fig. 3b). These monosaccharides could be attributed to the O-antigen repeating unit. In addition, the fraction contained rhamnose (Rha), glucosamine (GlcN) and ribose (Rib) (Fig. 3b). Presence of GlcN and Rib could result from contaminations with lipid A and nucleic acids. Rhamnose was shown to be part of the LOS core oligosaccharide of *C. canimorsus* 5^49^.

Altogether these data provide an additional evidence that Cc5 CPS and LOS O-antigen share common structural elements and epitopes. Furthermore, the finding of FucN, QuiN and an uronic acid is in perfect agreement with the annotation of the genes hit in the transposon mutants Y1C12, Y1D1 and Y3A2 (Fig. 2c and d).

### Generation of a non-capsulated *C. canimorsus* strain

As previously mentioned, the Y1C12 strain^13^ as well as all the transposon mutants affected in O-antigen and capsule synthesis were found to be more sensitive to the bactericidal effect of 10% human serum. Furthermore, the Y1C12 mutant was found to be also more sensitive to phagocytosis by polymorphonuclear leukocytes^13^. We thus asked whether these phenotypes would be the result of altered LOS O-antigen or absence of the CPS. To discriminate the contribution of these two structural elements, we needed to generate a strain lacking only the CPS and another strain lacking only the LOS-O-antigen (rough LOS, but encapsulated).

**Figure 4:**
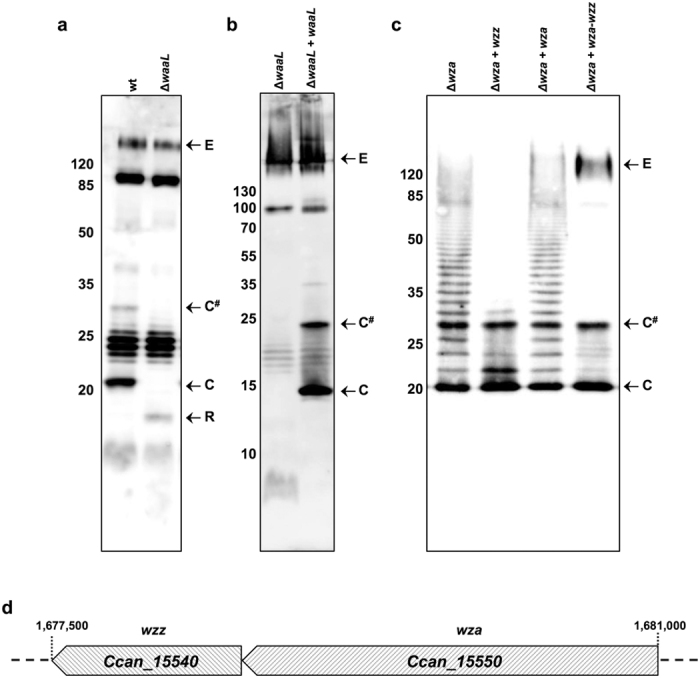
Generation of rough LOS and non-capsulated Cc5. (**a**) Immunoblot analysis of proteinase K-treated Cc5 wt and *waaL* mutant using anti-Cc5 serum. (**b**) Immunoblot analysis using Y1C12-adsorbed anti-Cc5 serum of proteinase K-treated Cc5 *waaL* mutant complemented or not with *waaL.* (**c**) Immunoblot analysis using Y1C12-adsorbed anti-Cc5 serum of proteinase K-treated Cc5 *wza* mutant complemented or not with *wzz, wza* or *wza* and *wzz*. (**d**) Genetic organization of the *wza-wzz* gene cluster.

In general, once the O-antigen units have been assembled by the Wzy polymerase, they are ligated to the lipid A-core by the WaaL ligase (formerly RfaL) to form a complete LPS^59^. Since our previous results suggested that Cc5 CPS is not linked to lipid A, we thought to generate a rough LOS strain, lacking the O-antigen but still harboring the CPS, by deleting the O-antigen ligase WaaL. We thus first searched for a WaaL homolog in Cc5 and found that the protein encoded by locus *Ccan_15430* has sequence similarity to known WaaL sequences. As anticipated, deletion of *Ccan_15430* determined the loss of wt LOS and the formation of a low molecular weight band likely representing the lipid A-core or rough LOS (Fig. 4a and b) thus suggesting that the mutated gene encodes for the O-antigen ligase WaaL of *C. canimorsus*. As expected, a normal capsular polysaccharide could be detected in the *waaL* mutant indicating that the absence of the O-antigen ligase affects only the LOS synthesis without interfering with the CPS assembly (Fig. 4a and b) and so reinforcing the evidence that Cc5 CPS is not linked to the lipid A-core. How the Cc5 CPS is linked to the bacterial surface was not investigated in detail and thus the presence of another lipid moiety anchoring the CPS to the bacterial surface could not be excluded. By deleting WaaL we thus obtained an encapsulated *C. canimorsus* harboring a rough LOS, lacking the O-chain.

We then aimed at generating a non-capsulated *C. canimorsus* strain, still endowed with its normal LOS (band C and C^#^). So far, our data indicated that Cc5 capsular polysaccharide is a group I (Wzy-dependent) CPS. The characteristic of the group I CPS assembled by a Wzy-dependent pathway is that single repeating units are synthesized and exported to the periplasm where the Wzy polymerase assembles them forming the CPS. The CPS assembly is controlled by the activities of the Wzc and Wzb proteins and the transport of the CPS to the bacterial surface occurs thanks to a lipoprotein named Wza that forms a multimeric translocation channel in the outer membrane^33^. Since deletion of Wza generally abolishes capsule translocation at the bacterial surface^46^, we searched in Cc5 genome for a homolog of *wza*. The protein encoded by gene *Ccan_15550* has 22% identity with the *E. coli* Wza protein. Deletion of *Ccan_15550* turned out to be polar on the downstream *Ccan_15540,* a *wzz* homolog, which regulates the length of the LPS O-chain^33^,^60^. We thus investigated the role of Wzz (Ccan_15540) and found that, unlike what happens in *E. coli*, in *C canimorsus*, it would control the length of the CPS rather than the LOS (Supplementary Data S3 and Supplementary Fig. S3).

Thus, since deletion of *wza* had a polar effect on *wzz*, in order to generate a non-capsulated *C. canimorsus* strain endowed with normal LOS, we expressed in trans the cloned *wzz* gene in the *wza* mutant and analyzed the LOS and CPS profiles. As shown in Fig. 4c, expression of *wzz* in the *wza* deletion strain restored the normal LOS profile but not the CPS indicating that *C. canimorsus* CPS is transported by the Wza protein. Finally, the *wza* mutant expressing *wzz* in trans turned out to be the required non-capsulated *C. canimorsus* strain that harbors normal LOS.

### The capsular polysaccharide contributes to survival in human serum, resistance to antimicrobial peptides and phagocytosis

**Figure 5:**
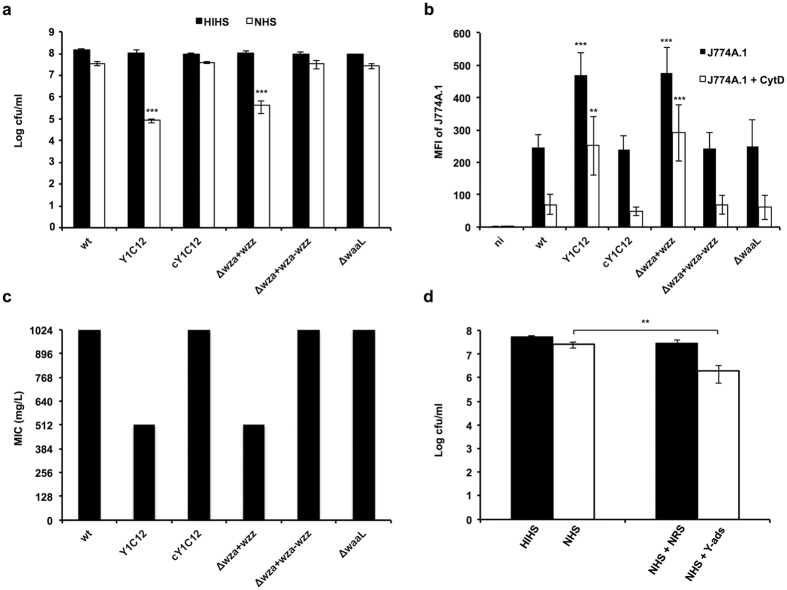
The capsule protects Cc5 against serum and polymyxin B killing and phagocytosis. (**a**) Total bacterial CFU present after incubation with 10% heat inactivated human serum (HIHS) or 10% normal human serum (NHS) for 1 hour at 37 °C. Mean values from 3 different experiments and standard deviations are represented (***p ≤ 0.001 compared to wt). (**b**) Phagocytosis of Cc5 bacteria by J774A.1 macrophages. J774A.1 macrophages were incubated with eFluor^®^-stained bacteria and subsequently analyzed by FACS. Cytochalasin D (CytD) pretreatment of macrophages was performed to inhibit phagocytosis. Mean values from 3 different experiments and standard deviations are represented (***p ≤ 0.001, **p ≤ 0.01 compared to wt). ni = non-infected macrophages. (**c**) Role of capsular polysaccharide on polymyxin B resistance. MIC of Polymyxin B for Cc5 wt, Y1C12, *waaL* (rough LOS), and Δ*wza* (*wzz*^+^) (non-capsulated) bacteria. Data were combined from 3 independent experiments where measured MIC were identical. (**d**) Effect of opsonization on serum killing. Total bacterial CFU of wt Cc5 present after incubation with HIHS or NHS and NHS in the presence of heat inactivated normal rabbit serum (NHS + NRS) or heat inactivated anti-Cc5 Y1C12–adsorbed rabbit serum (NHS + Y-ads). Mean values from 3 different experiments and standard deviations are represented (**p ≤ 0.01 compared to NHS).

In order to clarify the contribution of the LOS and the CPS in conferring resistance to the bactericidal activity of human serum, we compared the survival of wt and mutant bacteria lacking either the LOS O-antigen (Δ*waaL*), the CPS (Δ*wza, wzz*^+^) or both structures (Y1C12) in 10% human serum. As shown in Fig. 5a, the lack of the CPS only (Δ*wza, wzz*^+^) or of both CPS and LOS (Y1C12) determined a reduction of around 2.5 logs of the bacterial number thus suggesting a main role of the CPS in protecting against the bactericidal effect of human serum. Furthermore, encapsulated bacteria having a rough LOS (Δ*waaL*) were found to be as resistant as the wt strain thus indicating that the capsule alone is sufficient to confer resistance to human serum.

In order to determine the role of LOS O-antigen and CPS in resistance to phagocytosis, we then compared the same strains for phagocytosis by murine J774A.1 macrophages as determined by flow cytometry. With this method we assessed the mean fluorescence intensity (MFI) of macrophages incubated with fluorescently labeled bacteria. As shown in Fig. 5b, the lack of CPS (Δ*wza, wzz*^+^) or of both CPS and LOS resulted in an increase of about 2 fold of the MFI suggesting a main role of this structure in protecting against phagocytosis. In contrast, encapsulated bacteria lacking the LOS O-antigen (Δ*waaL*) were phagocytozed to the same extent as the wt strain thus excluding a main contribution of the LOS O-chain in preventing phagocytosis by macrophages (Fig. 5b).

Furthermore, we found that in the presence of Cytochalasin D, which inhibits phagocytosis^61^, the amount of bacteria associated to the macrophages was not identical for all strains tested. In particular, we observed an increase of 3 to 4 fold of the mean fluorescent intensity (MFI) of macrophages that were incubated with non-capsulated bacteria (Δ*wza, wzz*^+^) as compared to that of macrophages that were incubated with wt and *waaL* mutant bacteria (Fig. 5b). Since phagocytosis was inhibited, an increased MFI means a higher number of bacteria associated to the macrophage surface. It thus seems that bacteria lacking the CPS adhere more to the macrophages, which then might lead to an increased phagocytosis.

Overall these data indicate that *C. canimorsus* CPS could confer protection from phagocytosis by reducing bacterial adhesion to the macrophages.

Capsular polysaccharides have also been shown to play a role in protecting bacteria against antimicrobial peptides and proteins that are part of the innate immune response against bacteria^62^. Recently we showed that Cc5 bacteria are highly resistant to polymyxin B (MIC ≥1024 mg/L) (Fig. 5c and ref. 63). A main contribution to this property could be attributed to a specific feature of the lipid A moiety, which did neither affect the LOS O-antigen nor CPS expression^63^. In addition to the role of Cc5 lipid A modification we wondered whether the capsular polysaccharide and the LOS O-chain would contribute to protect bacteria against cationic antimicrobial peptides (CAMPs). We thus determined the resistance to polymyxin B of non-capsulated and rough LOS mutants. As shown in Fig. 5c, bacteria lacking the CPS only (Δ*wza, wzz*^+^) or both CPS and LOS (Y1C12) had a reduced MIC (≥512 mg/L) as compared to the wt (≥1024 mg/L). In contrast, encapsulated bacteria lacking the LOS O-antigen (Δ*waaL*) were found to be as resistant as the wt strain. These data thus indicate that the CPS structure is contributing to the protection against the bactericidal effect of polymyxin B and hence, likely of CAMPs.

In conclusion, our data indicate that the capsular polysaccharide of Cc5 likely contributes to the pathogenesis by providing protection against the bactericidal effect of human serum, antimicrobial peptides and phagocytosis by innate immune cells.

### Opsonization with anti-LOS O-antigen and capsule antibodies enhances killing in human serum

Opsonization plays a major role for effective killing of bacterial pathogens by human serum. We thus tested whether opsonization with the anti-LOS O-chain and capsule specific antibodies from the Y1C12-adsorbed antiserum would increase the bactericidal effect of human serum on *C. canimorsus*. As shown in Fig. 5d, in the presence of Y1C12-adsorbed antiserum the bactericidal effect of human serum on wt *C. canimorsus* was significantly increased (around 10 times) if compared to bacteria incubated in the human serum only. Taken together these data suggest that opsonization with LOS O-chain and capsule specific antibodies reduces the survival of *C. canimorsus* in human serum and thus indicate that these antibodies could have a potential protective role for the human host by increasing the ability of the innate immune system to clear the bacterial infection at its onset.

### A capsular polysaccharide is likely a commonality in *C. canimorsus*

The finding of a capsular polysaccharide in Cc5 and its likely role in resistance to the innate immunity raises the question whether other strains of *C. canimorsus* would be endowed with a similar structure at their surface.

**Figure 6:**
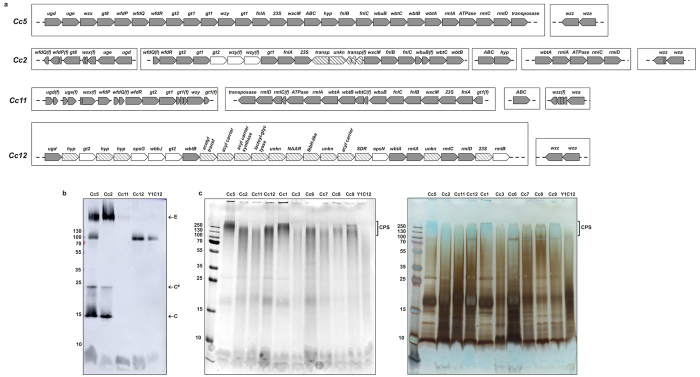
A capsular polysaccharide is present in several *C. canimorsus* isolates. (**a**) Comparison of the LOS/CPS-biosynthesis and transport loci of the four sequenced *C. canimorsus* strains. The boxes indicate different genomic loci. Orthologs of the Cc5 genes are indicated in grey. Genes indicated in white are strain specific genes likely involved in LOS/CPS biosynthesis. The hatched pattern indicates genes likely unrelated to LOS/CPS biosynthesis and transport. Fragmented genes are marked with (f). Note that the genomes of Cc2, Cc11 and Cc12 are draft genomes. For the sake of simplicity genes are not represented to scale. (**b**) Immunoblot analysis of proteinase K-treated Cc5, Cc2, Cc11 and Cc12 using Y1C12-adsorbed anti-Cc5 serum. **(c)** Alcian blue staining before (left) and after silver staining (right) of proteinase K-resistant structures of several *C. canimorsus* strains showing the presence of a capsular polysaccharide (CPS).

We first investigated the three *C. canimorsus* draft genomes available to date, namely *C. canimorsus* 2 (Cc2), *C. canimorsus* 11 (Cc11) and the *C. canimorsus* type strain ATCC35979 (Cc12)^64^. The three genomes contain *wza* and *wzz* orthologs organized in a two gene operon (Fig. 6a) suggesting the presence of a capsule in all these strains. As shown in Fig. 6a, we could also identify in all three genomes clusters of genes likely involved in LOS/CPS biosynthesis. However, while Cc2 and Cc11 encode orthologs for most of the Cc5 genes (Fig. 6a and Supplementary Table S3), albeit organized in several clusters distributed throughout the genomes, Cc12 showed a unique gene cluster but highly divergent from the Cc5 one in terms of composition (Fig. 6a). Then, we tested Cc2, Cc11 and Cc12 with the Cc5 LOS/CPS specific antiserum, As shown in Fig. 6b, the CPS and LOS from Cc2 were recognized suggesting that the O-antigen and capsule of Cc2 and Cc5 could have common epitopes. In contrast, the CPS and LOS from Cc11 and Cc12 were not recognized and hence could be substantially different from the Cc5 one, which was somehow expected for Cc12 given the low conservation of the LOS/CPS loci (Fig. 6a) but more surprising for Cc11. However, the Cc11 genome does not encode the ortholog of Ccan_23210 (Fig. 6a and Supplementary Table S3), a glycosyltransferase, shown to be crucial for Cc5 LOS and CPS biosynthesis (Supplementary Data S1 and Supplementary Fig. S2). Finally, in order to see if the presence of a CPS is a common feature of the *C. canimorsus* species, we analysed the polysaccharides of nine strains, including Cc2, Cc11 and Cc12, by SDS-PAGE followed by alcian blue and silver staining. As shown in Fig. 6c, all tested strains exhibited a capsular-like polysaccharide. In conclusion, the CPS is very likely a common feature of *C. canimorsus* but its composition and/or structure could vary between strains.

## Discussion

Here we present the first evidence of a capsular polysaccharide at the surface of the emerging human pathogen *C. canimorsus*. Although most of the work was done with strain Cc5, we show that the presence of a capsule is most likely a commonality in *C. canimorsus*. We provide genetic and analytical data showing that the CPS and LOS O-antigen repeating units share similar monosaccharides and epitopes. In the genome of strain Cc5, the genes encoding the O-chain and CPS repeating units biosynthesis, assembly and transport are grouped in a 27 kb cluster. This cluster includes the genes coding for the synthesis of UDP-GalA (*ugd* and *uge*), FucNAc (*fnlA, fnlB, fnlC*) and QuiNAc (*wbtA* and *wbtB*). In agreement with the genetic annotation we identified by GC-MS analysis as constituents of the Cc5 CPS and O-antigen units: QuiN, FucN, Glc, Gal, GalN and uronic acid. In addition, homologs of genes found in the O-antigen clusters of several *E. coli* serotypes as *wfdP, wfdQ* and *wfdR*^65^ are also present in the Cc5 O-antigen cluster. The cluster includes also genes involved in the synthesis of rhamnose (*rmlA, rmlC* and *rmlD*) that was previously shown to be part of the Cc5 core oligosaccharide^49^. Several putative glycosyltransferase genes are also present in the locus, including *wbuB* encoding a FucNAc transferase as well as Ccan_23210 and Ccan_23290 found to be crucial for the O-chain and CPS synthesis. Deletion of the genes involved in the synthesis or transfer of QuiNAc, FucNAc and GalA led to LOS O-chain modification and lack of CPS thus confirming that both O-antigen and CPS contain these monosaccharides or derivatives thereof and therefore likely share identical residues. Regarding O-units transport and assembly, the cluster encodes the Wzx flippase and the Wzy polymerase. By deleting the Wzx and Wzy homologs we could show that Cc5 harbors a LOS and that the CPS only is assembled by the Wzy polymerase.

Capsular polysaccharides play a role in the pathogenicity of several bacteria. In particular, in the phylum Bacteroidetes the presence of a capsule has been linked to the virulence of the human pathogens *Bacteroides fragilis* and *Porphyromonas gingivalis*. In *B. fragilis* several capsular polysaccharides have been identified and characterized^66^ and their role in pathogenicity has been linked to the ability of capsulated bacteria to induce abscess formation in animals^67^ and the capsule has been shown to mediate resistance to complement-mediated killing and to phagocytosis^68^. Interestingly, while in the genome of *Bacteroides fragilis* NCTC 9343 ten loci have been predicted to be implicated in polysaccharide biosynthesis, each including putative Wzx and Wzy homologs^69^, in the genome of four strains of *C. canimorsus*^51^,^64^ we could, so far, identify only one locus encoding the genes involved in both O-chain and CPS biosynthesis. Furthermore, *B. fragilis* are endowed with a so called micro-capsule (MC) (around 35 nm in size) whose variable expression is controlled by genes that are switched on and off by the site-specific inversion of promoter sequences^70^,^71^,^72^. Such sequences could not be found in the genome of Cc5^51^ either, suggesting that CPS expression is regulated differently in the two species. In addition, *B. fragilis* harbor antigenically distinct and within-strain variable large capsules (LCs) and small capsules (SCs)^52^,^73^. In contrast in *C. canimorsus*, electron microscopy observation revealed the presence of a 50 nm in size CPS that was completely absent in the O-unit biosynthesis mutant thus suggesting that *C. canimorsus* harbors only one CPS type, related to the LOS O-antigen.

In *P. gingivalis* the capsule has been shown to be a major virulence factor in a mouse abscess model and to play a role in decreasing the host inflammatory response and phagocytosis^74^. In our previous work we showed that Cc5 is resistant to the bactericidal effect of 10% human serum and is able to escape phagocytosis^13^. Here we show that the presence of the CPS increases Cc5 survival in human serum, resistance to CAMPs and reduces the uptake by macrophages. We found this latter effect to be a consequence of increased adhesion of bacteria lacking the CPS to macrophages thus suggesting that the CPS reduces the ability of *C. canimorsus* to adhere to host cells. Unfortunately, to date, no reliable animal model exists for *C. canimorsus* infections and thus the role of the CPS *in vivo* could not be determined. Nevertheless our *in vitro* results clearly suggest that the CPS could play a major role in *C. canimorsus* infections probably at their onset conferring protection against the bactericidal effect of serum and phagocytosis. Furthermore, we could show that antibodies targeting the O-chain and the CPS increase the killing of Cc5 in human serum thus suggesting a potential protective role for the host against these tremendous bacterial infections.

## Materials and Methods

### Bacterial strains and growth conditions

Bacterial strains used in this study are listed in Supplementary Table S1. *C. canimorsus* were routinely grown on heart infusion agar (Difco) supplemented with 5% sheep blood (Oxoid) plates (SB plates) for 2 days at 37 °C in the presence of 5% CO_2_. *E. coli* strains were routinely grown in lysogeny broth (LB) at 37 °C. As selective agents, antibiotics were added at the following concentrations: 100 μg/ml ampicillin (Amp), 50 μg/ml kanamycin (Km) for *E. coli* and 10 μg/ml erythromycin (Em), 10 μg/ml cefoxitin (Cfx), 20 μg/ml gentamicin (Gm), 10 μg/ml tetracycline (Tc) for *C. canimorsus*.

### *C. canimorsus* 5 and Y1C12-adsorbed antisera

Polyclonal serum against *C. canimorsus* 5 was generated from a rabbit by immunization with paraformaldehyde fixed *C. canimorsus* 5 (Animal facility of the University of Namur). The Y1C12-absorbed serum was prepared by incubating three times an excess amount of paraformaldehyde fixed Y1C12 mutant bacteria harvested from SB plates and washed in phosphate-buffered saline (PBS) with anti-*C. canimorsus* 5 serum at 4 °C for minimum 4 hours. Bacteria were removed by repeated centrifugation.

### Immunoblotting of proteinase K-resistant structures

Bacteria grown for 2 days at 37 °C with 5% CO_2_ were harvested from SB plates, washed once in 1 ml of PBS and adjusted to an OD_600_ of 1 in PBS. 750 μl bacterial suspension was pelleted and resuspended in 125 μl ddH_2_O loading buffer (1% sodium dodecyl sulfate [SDS], 10% glycerol, 50 mM dithiothreitol, 0.02% bromophenol blue, 45 mM Tris (pH 6.8)). Samples were heated at 99 °C for 10 minutes. Proteinase K (50 μg/ml final concentration) was added and samples were incubated at 37 °C overnight. After incubation samples were heated again for 10 minutes at 99 °C and a second volume of proteinase K (equal to the first) was added. Samples were incubated at 55 °C for 3 hours, heated again for 5 minutes at 99 °C and loaded on a 15% SDS-PAGE. Samples were analyzed by western blotting.

### *In vivo* radiolabeling with [^3^H] palmitate and fluorography

Bacteria grown for 2 days on SB plates were collected and resuspended in PBS to an OD_600_ of 0.1. Six μl of bacterial suspension (approx. 3 × 10^5^ bacteria) were seeded in 3 ml of DMEM (41965-039; Gibco) containing 10% heat-inactivated human serum (HIHS) in 12-well plates (665 180; Greiner Bio-one). After 18 h of incubation at 37 °C with 5% CO_2_, [9,10-^3^H] palmitic acid (32 Ci/mmol; NET043; Perkin-Elmer Life Sciences) was added to a final concentration of 50 μCi/ml and incubation was continued for 6 h. Bacteria were then collected by centrifugation, washed 2 times with 1 ml PBS and pellets were stored at −20 °C until further use. Pellets were treated with proteinase K as described above. Samples were loaded on 12% SDS PAGE gels and after electrophoresis, gels were fixed in 25:65:10 isopropanol:water:acetic acid solution overnight and subsequently soaked for 30 min in Amplify (NAMP100; Amersham) solution. Gels were vacuum dried and exposed to SuperRX autoradiography film (Fuji) for 12–18 hours until desired signal strength was reached.

### Gene annotation and LOS/CPS loci analysis

BlastP^75^ and Delta Blast searches were carried out against the *C. canimorsus* 5 genome^51^. Query sequences were obtained from the NCBI database. All available search sequences for a corresponding gene of interest were used. The highest scoring subjects over all the searches have been annotated as corresponding enzymes. Analysis of synteny of the LOS/CPS *C. canimorsus* loci was obtained from the MicroScope Microbial Genome Annotation and Analysis Platform (https://www.genoscope.cns.fr/agc/ microscope/home/index.php)^76^. Putative orthologous relations between two genomes are defined as gene couples satisfying the bi-directional best hit (BBH) criterion or a BlastP alignment threshold, a minimum of 35% sequence identity on 80% of the length of the smallest protein. These relations are subsequently used to search for conserved gene clusters, synteny groups (syntons), among several bacterial genomes. All possible kinds of chromosomal rearrangements are allowed (inversion, insertion/deletion).

### Mutagenesis and allelic exchange

Genetic manipulations of *C. canimorsus* 5 wt have been previously described^77^. Briefly, replacement cassettes with flanking regions spanning approximately 500 bp homologous to direct target gene framing regions were constructed with a three-fragment overlapping-PCR strategy. First, two PCR were performed on 100 ng of Cc5 genomic DNA with primers A and B (Supplementary Table S2) for the upstream flanking regions and with primers E and F for the downstream regions. Primers B and E contained an additional 5′ 20-nucleotide extension homologous to the *ermF* or *tetQ* insertion cassettes. The *ermF* and *tetQ* resistance cassettes were amplified from plasmids pMM13 and pMM104.A^77^ DNA respectively with primers C and D. All three PCR products were cleaned and then mixed in equal amounts for PCR using Phusion polymerase (Finnzymes). The initial denaturation was at 98 °C for 2 min, followed by 12 cycles without primers to allow annealing and elongation of the overlapping fragments (98 °C for 30 s, 50 °C for 40 s, and 72 °C for 2 min). After the addition of external primers (A and F), the program was continued with 20 cycles (98 °C for 30 s, 50 °C for 40 s, and 72 °C for 2 min 30 s) and finally 10 min at 72 °C. Final PCR products were then digested with *Pst*I and *Spe*I for cloning into the appropriate sites of the *C. canimorsus* suicide vector pMM25^77^. Resulting plasmids were transferred by RP4-mediated conjugative DNA transfer from *E. coli* S17-1 to *C. canimorsus* 5 to allow integration of the insertion cassette. Transconjugants were then selected for the presence of the *ermF* or *tetQ* resistance cassettes on erythromycin and tetracycline plates respectively and checked for sensitivity to cefoxitin. Deletion of the appropriate regions was verified by PCR.

### Construction of plasmids for trans-complementation

Full length *C. canimorsus* 5 genes: *Ccan_23200 (wzx), Ccan_23280 (wzy), Ccan_15430 (waaL), Ccan_23190 (uge), Ccan_23400 (wbtA), Ccan_15540 (wzz), Ccan_15550 (wza), Ccan_23210 and Ccan_23290*, were amplified from *C. canimorsus* 5 genomic DNA using primers listed in Supplementary Table S2 and cloned into plasmid pPM5^78^ into *Nco*I and *Xho*I restriction sites leading to plasmids listed in Supplementary Table S1. Gene couples *Ccan_15540–15550, Ccan_23200–23210* and *Ccan_23280–23290* were co-amplified from *C. canimorsus* 5 genomic DNA and cloned into plasmid pPM5^78^ into *Nco*I and *Xho*I restriction sites.

### Human serum sensitivity assay

Bacteria were harvested by gently scraping colonies off the agar surface. They were washed and resuspended in PBS to an OD_600_ of 1. Normal human serum (NHS) from healthy volunteers was pooled, aliquoted, and stored at −80 °C. Serum was heat inactivated (HIHS) at 55 °C for 30 min when indicated. A total of around 1 × 10^7^ bacteria were incubated for 1 or 3 hours in 200 μl 10% NHS or HIHS in PBS (around 5 × 10^7^ bacteria/ml) at 37 °C with 5% CO_2_. Serial dilutions were plated onto SB plates, and viable colonies were counted after 48 hours of incubation. To test the impact of anti-LOS and capsule antibodies on the survival of *C. canimorsus* 5, wt bacteria were treated as described above (1 hour incubation) with the exception that heat inactivated Y1C12-adsorbed anti Cc5 serum (1/200 dilution) or heat inactivated normal rabbit serum (1/200 dilution) were added to the bacterial suspensions before incubation.

### Polymyxin B sensitivity assay

Polymyxin B sulphate was obtained from Sigma-Aldrich. The agar dilution method was performed based on the CLSI/NCCLS recommendations^79^. Briefly, 10^4^ bacteria diluted in PBS were spotted in 2 μl on SB plates containing Polymyxin B ranging from 0.5 mg/L to 1024 mg/L (2-fold increase per condition). Plates were examined for growth of colonies after 48 hours and 72 hours.

### Phagocytosis assay by flow cytometry

Murine J774A.1 (ATCC TIB-67) macrophages were cultivated in RPMI 1640 (Gibco) medium supplemented with 2 mM glutamine (Gibco) and 10% fetal bovine serum (Gibco). For phagocytosis assay macrophages were seeded overnight in 24 well plates (1 × 10^5^ cells/well) at 37 °C and 5% CO_2_. To inhibit phagocytosis macrophages were treated prior to infection for 30 min with 10 μM cytochalasin D (Sigma Aldrich). Bacteria (OD_600_ of 1) were stained with eFLuor^®^ 670 (eBioscience) at a concentration of 5 μM for 15 min at 37 °C in PBS then washed 3 times in PBS. Macrophages were infected at a MOI of 10 with labeled bacteria, spun (400 × g, 5 min) and incubated for 1 h at 37 °C and 5% CO_2._ Macrophages were then washed with PBS, scraped and washed again 2 more times with PBS before being fixed for 15 min in 4% PFA (Sigma Aldrich). Macrophages were then analyzed by FACS on a FACSCalibur using Cell quest Pro (BD Bioscience).

### Statistical analysis

Statistical analyses were realized using the software GraphPad Prism by one-way ANOVA test followed by a Bonferroni test.

### LOS isolation

*C. canimorsus* were harvested from 600 SB plates in PBS and washed with ddH_2_O, ethanol (300 ml) and acetone (300 ml), followed each time by centrifugation at 18000 × g for 30 min. LOS used for western blot analysis was isolated from *C. canimorsus* 5 wt and Y1C12 mutant as described in ref. 36.

### Alcian blue and silver staining of proteinase K-resistant structures

Bacteria treated with proteinase K as described above were loaded on 15% Tris-glycine SDS polyacrylamide gels. Following electrophoresis, the gels were stained first with Alcian blue (Sigma) (0.125% Alcian blue in 40% ethanol–5% acetic acid) and then with silver as described in ref. 80.

### Preparation of the CPS and O-antigen for composition analysis

*C. canimorsus* were harvested from 25 SB plates in PBS with 1% phenol and washed twice with ddH_2_O. The isolation of LOS was achieved after phenol-water extraction^57^ with or without phase separation. The lyophilized extracts were analyzed by western blot using full and Y1C12-adsorbed anti-Cc5 serum before performing chemical modifications and carbohydrate analysis. Crude extracts were hydrolyzed with 5% AcOH (100 °C, 2 hours), cooled, lyophilized, resuspended in 2 ml of water, centrifuged and fractionated on a Sephadex S-300 column. LMW fractions were pooled and re-fractionated on a Biogel P2 column.

### General and analytical methods

Gel filtration chromatography was performed on a Sephacryl S-300 column (Pharmacia, 1 × 80 cm) and a Bio-Gel P2 column (1.6 × 80 cm) eluted with 0.1% acetic acid. Aliquots of each fraction were assayed for neutral sugars^81^. Monosaccharides were identified by GC-MS with a Trace GC ULTRA system (Thermo Scientific) equipped with an NMTR-5MS capillary column (30 m × 0.25 mm) with a temperature gradient of 170 °C (3 min) to 250 °C at 5 °C min^−1^ and with a DSQ II MS detector. Prior to analysis, carbohydrate samples were hydrolyzed with 4 M trifluoroacetic acid (110 °C, 3 h) and converted to alditol acetates by conventional methods. For the identification of QuiNAc and FucNAc, authentic standards of *P. aeruginosa* PA14 and PAO1 O-antigens were used.

### Transmission electron microscopy (TEM)

#### Thin sectioning

Bacteria were harvested from SB plates and resuspended in PBS to an OD_600_ of 1 (corresponding to 5 × 10^8^ bacteria/ml). Around 3 × 10^5^ bacteria were seeded in 1 ml of DMEM (41965-039; Gibco) containing 10% heat-inactivated human serum (HIHS) in 12-well plates (665 180; Greiner Bio-one) and incubated 23 hours at 37 °C with 5% CO_2_. 7 ml of Cc5 wt and Y1C12 cultures were centrifuged 5 min at 5000 g and washed with 10 ml PBS. Bacteria were then centrifuged and resuspended in 5 ml PBS containing 2% glutaraldehyde. Bacteria were then fixed in Karnofski solution (3% paraformaldehyde, 0.5% glutaraldehyde in 10 mM PBS, pH 7.4) for 1 hour, washed once in PBS and post-fixed first in 1% reduced osmium tetroxide (containing 1.5% potassium ferricyanide) for 40 minutes and subsequently in 1% osmium tetroxide for another 40 minutes. After washing in water, fixed samples were dehydrated, embedded in Epon resin, and processed for TEM as described^82^. EM micrographs were recorded on CCD camera using a Morgani transmission electron microscope (FEI, The Netherlands) operating at an acceleration voltage of 80 kV.

#### Cryo-transmission electron microscopy (Cryo-TEM) analysis

4 μl of bacteria resuspended in a PBS 2% glutaraldehyde solution were adsorbed onto glow-discharged holey carbon-coated grid (quantifoil, Germany) plunge-frozen into liquid ethane at −178 °C using a Leica EM GP machine (Leica Microsystems, Austria). Frozen grids were transferred onto a Philips CM200-FEG electron microscope using a Gatan 626 cryo-holder. Electron micrographs were recorded at an accelerating voltage of 200 kV and a nominal magnification of 50,000x, using a low-dose system (10 e-/Å^2^) and keeping the sample at −175 °C. Defocus values were −3 μm. Micrographs were recorded at 4 K × 4 K CMOS camera (TVIPS, Germany).

#### Ethics statement

This study was approved by the Comité d’éthique médicale from the CHU Mont-Godinne/Université de Louvain (no: B039201316262) and experiments carried out according to the declaration of Helsinki and the Belgian law on experiments in humans from March 7th, 2004. Human blood was collected from healthy volunteers who were informed about the aim of the study and the risks of blood withdrawal and had signed a written informed consent. The animal handling and procedures used in this study were approved by the Animal Welfare Committee of the University of Namur (no: FUNDP LE 08/106).

## Additional Information

**How to cite this article**: Renzi, F. *et al*. Evidence for a LOS and a capsular polysaccharide in *Capnocytophaga canimorsus.* *Sci. Rep.* **6**, 38914; doi: 10.1038/srep38914 (2016).

**Publisher's note:** Springer Nature remains neutral with regard to jurisdictional claims in published maps and institutional affiliations.

## Supplementary Material

Supplementary Information
