# Supplementary material for: Evidence for a LOS and a capsular polysaccharide in Capnocytophaga canimorsus
Source: Sci Rep. 2016 Dec 15;6:38914. doi: 10.1038/srep38914 (PMC5156936; doi:10.1038/srep38914)
Supplement: Supplementary Information [file srep38914-s1.pdf]

Supplementary Information

Evidence for a LOS and a capsular polysaccharide in *Capnocytophaga canimorsus*

Francesco Renzi, Simon J Ittig, Irina Sadovskaya, Estelle Hess, Frederic Lauber, Melanie Dol, Hwain Shin, Manuela Mally, Chantal Fiechter, Ursula Sauder, Mohamed Chami and Guy R Cornelis

Supplementary data

Data S1. The O-chain units are transported by Wzx

As shown in Supplementary Fig. S2, deletion of the putative *wzx* (*Ccan_23200*) determined the disappearance of the CPS and of normal LOS (band C) and the appearance of lower molecular weight LOS bands. However, trans complementation of *wzx* did not restore the wt LOS and CPS profiles but determined the formation of lower molecular weight LOS bands (Supplementary Fig. S2) thus suggesting a polar effect of the deletion on the downstream genes. Indeed, the downstream gene *Ccan_23210* encodes an A4GalT_like glycosyltransferase that could be responsible for the addition of galactose or glucose residues to the O-chain. We then co-expressed genes *Ccan_23200* (*wzx*) and *Ccan_23210* in trans in the *wzx* mutant strain and verified the LOS and CPS profiles. As shown in Supplementary Fig. S2, co-expression restored the wt phenotype thus confirming that deletion of *wzx* had

a polar effect on *Ccan_23210*. Then, in order to determine the role of *Ccan_23200*, we expressed *Ccan_23210* in trans in the *wzx*-deleted strain. Expression of *Ccan_23210* did not restore the wt phenotype (Supplementary Fig. S2) thus suggesting that *Ccan_23200* could indeed encode for the Wzx O-chain transporter.

Data S2. The capsule is polymerized by Wzy

Trans complementation of the *Ccan_23280* (*wzy*) mutant did not restore the wt LOS and CPS profiles thus suggesting a polar effect of the deletion on downstream genes (Supplementary Fig. S2). Indeed, the *wzy* downstream gene codes for a glycosyltransferase of the family 1 that could be involved in the biosynthesis of the O-chain. We then co-expressed genes *Ccan_23280* (*wzy*) and *Ccan_23290* in trans in the *wzy* mutant strain and verified the LOS and CPS profiles. As shown in Supplementary Fig. S2, co-expression of *Ccan_23280* and *Ccan_23290* restored the CPS and LOS profiles thus confirming that deletion of *wzy* had a polar effect on *Ccan_23290*. Then, in order to determine the role of *Ccan_23280*, we expressed *Ccan_23290* in trans in the *wzy* deletion strain. Expression of *Ccan_23290* restored the wt LOS profile but not the CPS (Supplementary Fig. S2.), suggesting that *Ccan_23280* indeed encodes the Wzy O-antigen polymerase that is responsible for the assembly of the CPS but not of the LOS O-chain.

Data S3. The capsule assembly is controlled by Wzz

The *Ccan_15550* (*wza*) deletion mutant lacked the CPS but surprisingly showed a ladder pattern of bands migrating higher than wt LOS (Fig. 4c), a

phenotype resembling the one observed for the *wzy* (*wzy*$^+$, *23290*$^+$) strain. We hypothesized that this ladder could be composed of LPS harboring O-chains of different lengths and that deletion of *Ccan_15550* somehow affected the regulation of the LOS O-antigen length.

The length of the LPS O-chain is known to be regulated by Wzz (formerly Cld or Rol) [33,60] and the LPS profiles of several bacteria lacking this protein have been shown to be a ladder of different molecular weight bands. *Ccan_15540*, located immediately downstream of *Ccan_15550* (*wza*) (Fig. 4c) is a *wzz* homolog and we thus hypothesized that the *wza* deletion could have a polar effect on *wzz*. To prove our hypothesis we generated a *wzz* (*Ccan_15540*) deletion strain and analyzed by western blot its polysaccharide structures. The *wzz* mutant displayed the same phenotype as the *wza* one, *i.e.* a ladder pattern of bands and absence of the capsular polysaccharide (Supplementary Fig. S3). The lack of the CPS in the *wzz* mutant was somehow unexpected but it could be explained either by exhaustion of O-chain repeating units that are linked to lipid A generating a ladder pattern of LPS, or either by a direct involvement of Wzz in the CPS assembly. In order to understand the role of Wzz, we generated a double *wzz* and *waaL* mutant strain and analyzed its polysaccharide structures. As shown in Supplementary Fig. S3, the double mutant lacked both CPS and the laddering of LPS thus indicating that it is not the formation of an LPS ladder that prevents capsule formation but that, in *C. canimorsus*, Wzz could have a direct role in CPS assembly, *i.e.* controlling the CPS length. This is also supported by the absence of a Wzc homolog in Cc5 genome and by the genetic organization of the *wza* and *wzz* genes encoded in one operon (Fig. 4d). In addition, in the *wzz* mutant, the intensity of LOS

bands C and $C^{\#}$ was stronger than the one of the LPS ladder (Supplementary Fig. S3) suggesting that formation of normal LOS is independent from Wzz but could be controlled by another, yet unidentified, ruler-like protein.

Supplementary figures

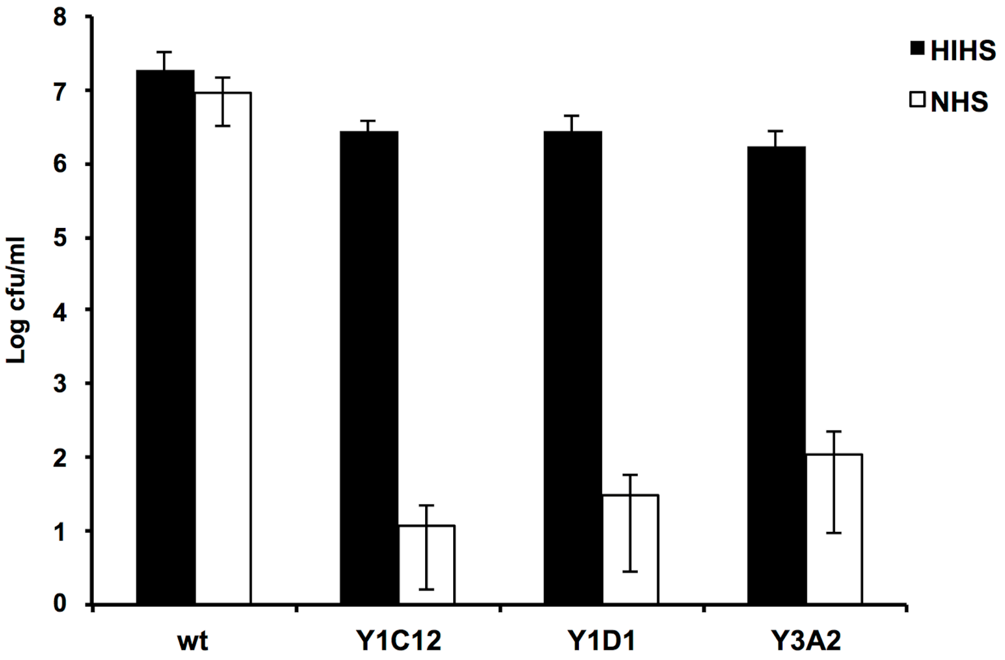

Figure S1. The transposon mutants are serum sensitive.

Total bacterial CFU present after incubation of wt or Tn4351 mutated Cc5 in 10% heat inactivated normal human serum (HIHS) or 10% normal human serum (NHS) for 180 min at 37°C. Mean values from 3 different experiments and standard deviations are represented.

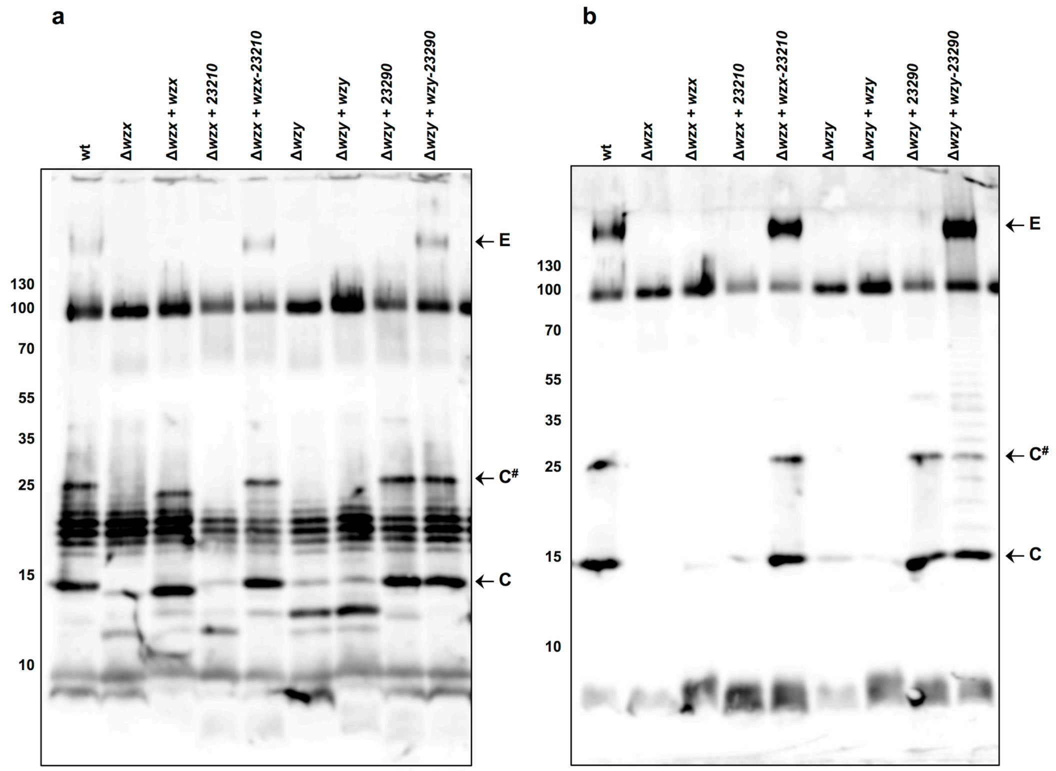

Figure S2. The Cc5 capsule is assembled via the Wzx/Wzy pathway.

(**a**) Immunoblot analysis of proteinase K-treated wt Cc5, *wzx* and *wzy* mutant strains complemented or not with different genes combinations using anti-Cc5 serum. (**b**) Immunoblot analysis as described for panel (**a**) using Y1C12-adsorbed anti-Cc5 serum.

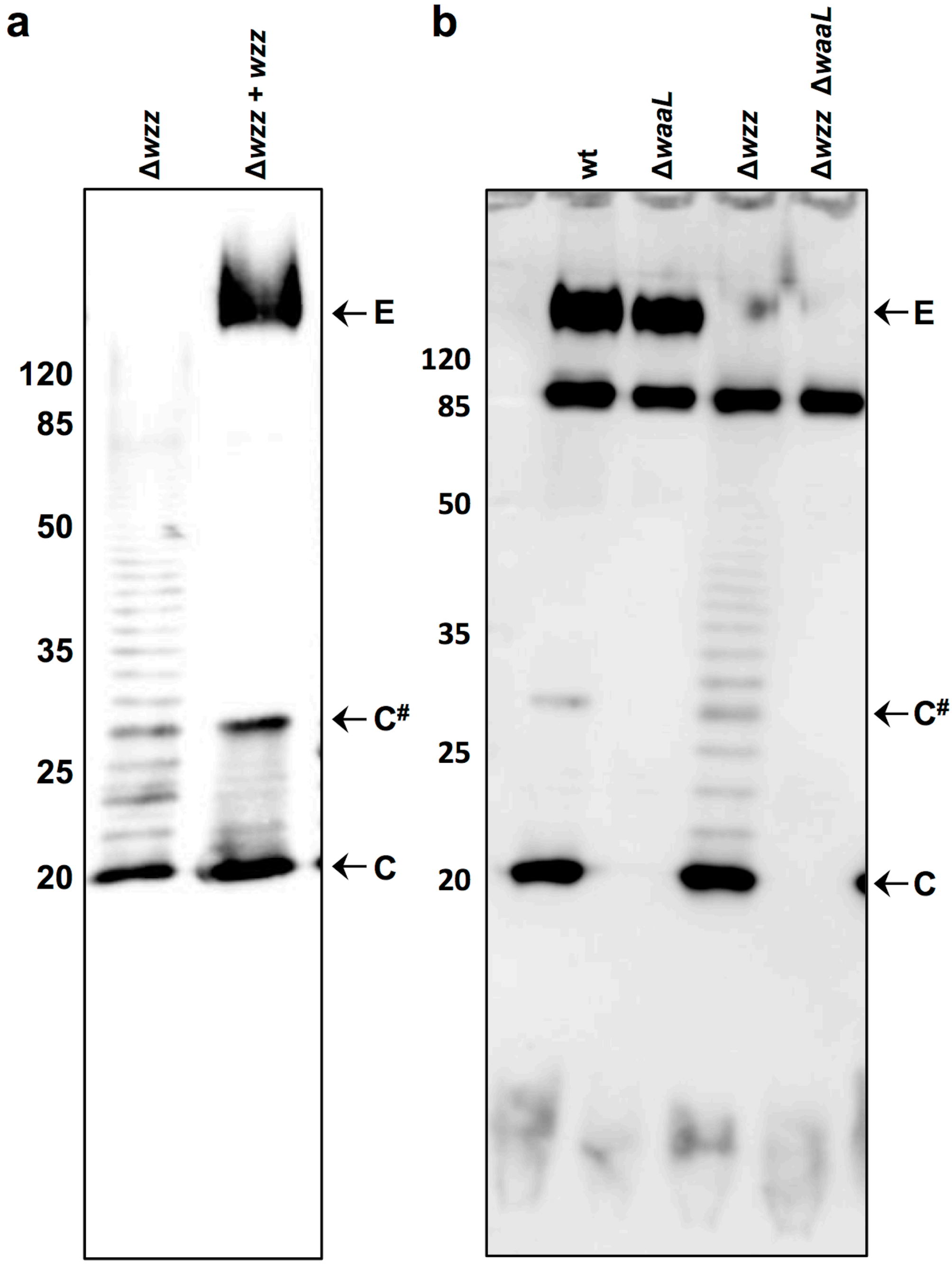

Figure S3. The CPS assembly is controlled by Wzz.

(**a**) Immunoblot analysis using Y1C12-adsorbed anti-Cc5 serum of proteinase K-treated Cc5 *wzz* mutant complemented or not with *wzz.* (**b**) Immunoblot analysis using Y1C12-adsorbed anti-Cc5 serum of proteinase K-treated Cc5 wt, *waaL*, *wzz* and *wzz waaL* double mutants.

Supplementary Tables

Table S1. Bacterial strains and plasmids used in this study

**Bacterial strains**	**Description or genotype**	**Reference**
E. coli		
Top10	F^{-} *mcrA* Δ(*mrr-hsdRMS-mcrBC*) ϕ80*lacZ*ΔM15 Δ*lacX*74 *recA1 araD139* Δ(*araleu*)*7697 galU galK rpsL endA1 nupG*; Sm^{r}	Invitrogen
S17-1	*hsdR17 recA1* RP4-2-*tet::*Mu1*kan::*Tn7; Smr	[83]
C. canimorsus		
	**Origin or genotype**	
C. canimorsus 5 (Cc5)	Human fatal septicemia after dog bite (1995). (BCCM- LMG 28512)	[14]
C. canimorsus 2 (Cc2)	Human septicaemia (1989).	
C. canimorsus 11 (Cc11)	BCCM/LMG11551, MCCM01373	
C. canimorsus 12 (Cc12)	Human septicaemia (1961). ATCC35979, CDC7120	
C. canimorsus 1 (Cc1)	Human infection (1985). BCCM/LMG 11511 CCUG 17234; strain P810;strain SSI P810	
C. canimorsus 3 (Cc3)	Human septicaemia (1990).	
C. canimorsus 6 (Cc6)	Human septicaemia (1996).	
C. canimorsus 7 (Cc7)	Human septicaemia (1998).	

C. canimorsus 8 (Cc8)	Human septicaemia (2004).	
C. canimorsus 9 (Cc9)	Human septicaemia (1965). BCCM/LMG11510, CCUG12569, CDCA3626	
Y1C12	*C. canimorsus* 5 *Ccan_23370*::Tn*4351*; Em^r	[13]
Y1D1	*C. canimorsus* 5 *Ccan_23400*::Tn*4351*; Em^r	This study
Y3A2	*C. canimorsus* 5 *Ccan_23190*::Tn*4351*; Em^r	This study
U5D4	*C. canimorsus* 5 *Ccan_23360*::Tn*4351*; Em^r	This study
Cc5 Δ*wza*	Replacement of *Ccan_15550* by *ermF* using primers 6541, 6542, 6543, 6544,6545,6546; Em^r	This study
Cc5 Δ*wzx*	Replacement of *Ccan_23200* by *ermF* using primers 7801, 7802, 7803, 7804, 7805; Em^r	This study
Cc5 Δ*wzy*	Replacement of *Ccan_23280* by *ermF* using primers 7910, 7911, 7912, 7913, 7914, 7915; Em^r	This study
Cc5 Δ*waaL*	Replacement of *Ccan_15430* by *ermF* using primers 7810, 7811, 7812, 7813, 7814, 7815; Em^r	This study
Cc5 Δ*wzz*	Replacement of *Ccan_15540* by *ermF* using primers 6547, 6548, 6549, 6550, 6551, 6552; Em^r	This study
Cc5 Δ*wzz* Δ*waaL*	Replacement of *Ccan_15430* by *tetQ* using primers 7810, 7813, 8136, 8137,8138, 8139 in the Δ*wzz* genetic background; Em^r; Tc^r	This study
Δ*waaL* + *waaL*	Cc5 Δ*waaL* strain complemented with plasmid pFR38	This study

Δ*wza* + *wzz*	Cc5 Δ*wza* strain complemented with plasmid pFR31	This study
Δ*wza* + *wza-wzz*	Cc5 Δ*wza* strain complemented with plasmid pFR33	This study
Δ*wza* + *wza*	Cc5 Δ*wza* strain complemented with plasmid pFR32	This study
Δ*wzz* + *wzz*	Cc5 Δ*wzz* strain complemented with plasmid pFR31	This study
cY1C12	Y1C12 strain complemented with plasmid pCF3.	[13]
cY1D1	Y1D1 strain complemented with plasmid pFR35	This study
cY3A2	Y3A2 strain complemented with plasmid pFR34	This study
Δ*wzx* + *wzx*	Cc5 Δ*wzx* strain complemented with plasmid pFR36	This study
Δ*wzx* + *23210*	Cc5 Δ*wzx* strain complemented with plasmid pFR39	This study
Δ*wzx* + *wzx-23210*	Cc5 Δ*wzx* strain complemented with plasmid pFR40	This study
Δ*wzy* + *wzy*	Cc5 Δ*wzy* strain complemented with plasmid pFR37	This study
Δ*wzy* + *23290*	Cc5 Δ*wzy* strain complemented with plasmid pFR41	This study
Δ*wzy* + *wzy-23290*	Cc5 Δ*wzy* strain complemented with plasmid pFR42	This study
**Plasmids**	**Description**	**Reference**
pPM5	Ori_{ColE1}, ori_{pCC7}, Ap^r ,Cf^r, *E. coli - C. canimorsus* shuttle expression vector.	[78]

pFR31	Full length *Ccan_15540* (*wzz*) amplified with primers 7793 and 7794 and cloned into pPM5 using *NcoI* and *XhoI* restriction sites.	This study
pFR32	Full length *Ccan_15550* (*wza*) amplified with primers 7791 and 7792 and cloned into pPM5 using *NcoI* and *XhoI* restriction sites.	This study
pFR33	Full length *Ccan_15550* (*wza*) and *Ccan_15540* (*wzz*) co-amplified with primers 7791 and 7794 and cloned into pPM5 using *NcoI* and *XhoI* restriction sites.	This study
pFR34	Full length *Ccan_23190* (*uge*) amplified with primers 7771 and 7772 and cloned into pPM5 using *NcoI* and *XhoI* restriction sites.	This study
pFR35	Full length *Ccan_23400* (*wbtA*) amplified with primers 7773 and 7774 and cloned into pPM5 using *NcoI* and *XhoI* restriction sites.	This study
pFR36	Full length *Ccan_23200* (*wzx*) amplified with primers 8234 and 8235 and cloned into pPM5 using *NcoI* and *XhoI* restriction sites.	This study
pFR37	Full length *Ccan_23280* (*wzy*) amplified with primers 8232 and 8233 and cloned into pPM5 using *NcoI* and *XhoI* restriction sites.	This study
pFR38	Full length *Ccan_15430* (*waaL*) amplified with primers 8236 and 8237 and cloned into pPM5 using *NcoI* and *XhoI* restriction sites.	This study
pFR39	Full length *Ccan_23210* amplified with primers 8243 and 8244 and cloned into pPM5 using *NcoI* and *XhoI* restriction sites.	This study
pFR40	Full length *Ccan_23200* (*wzx*) and *Ccan_23210* co-amplified with primers 8234 and 8244 and cloned into pPM5 using *NcoI* and *XhoI* restriction sites.	This study

pFR41	Full length *Ccan_23290* amplified with primers 8245 and 8246 and cloned into pPM5 using *NcoI* and *XhoI* restriction sites.	This study
pFR42	Full length *Ccan_23280* (*wzy*) and *Ccan_23290* co-amplified with primers 8232 and 8246 and cloned into pPM5 using *NcoI* and *XhoI* restriction sites.	This study
pMM104.1	ColE1 *ori* (pCC7 *ori*); Ap^r (Tc^r); *E. coli-C. canimorsus* shuttle plasmid, RP4 oriT. *Pst*I fragment of pMM47.A containing repA inserted into *PstI* site of pLYL001	[77]
pMM13	ColE1 *ori*; Apr (Em^r); *ermF* from pEP4351	[77]

Table S2. Oligonucleotides used in this study

Ref.	Name	Sequence 5'-3'	Restriction	Gene	PCR
6541	wza-A	ccctgcaggcctatatgcgtatggcac	PstI	*wza*	A
6542	wza-B	gagtagataaaagcactgttcgtttttatgtatttgggca		*wza*	B
6543	wza-C	gcactacatttgcccaaatacataaaaaacgaacagtgcttttatctactccgatagcttc		*ermF*	C
6544	wza-D	caatctcatctgtattactaactttattttcgcacataaactacgaaggatgaaatttttcagggacaac		*ermF*	D
6545	wza-E	aaaaatttcatccttcgtagtttatgtgcgaaaataaagttagtaatac		*wza*	E
6546	wza-F	caactagtaatgcaggaagccctatg	SpeI	*wza*	F
7793	wzz-fw	cataccatgggatgcgaaaataaagttagtaatac	NcoI	*wzz*	
7794	wzz-rev	ccgctcgagttaaactttttcagcttttaac	XhoI	*wzz*	
6547	wzz-A	cctggtcgacgcagctgatgacacagaat	PstI	*wzz*	A
6548	wzz-B	gagtagataaaagcactgttaaattacttaaatgtattgaataacaaaact		*wzz*	B

6549	wzz-C	caacaatgggagttttgttattcaatacatttaagtaatttaacagtgcttttatctactccgatagcttc		*ermF*	C
6550	wzz-D	actcaacaacttttaactttttgtatattcaaatgtcttaattctacgaaggatgaaatttttcagggacaac		*ermF*	D
6551	wzz-E	aaaaatttcatccttcgtagaattaagacatttgaatatacaaaaagttaaa		*wzz*	E
6552	wzz-F	caactagtcagcgcttatgccctta	SpeI	*wzz*	F
7791	wza-fw	cataccatgggatgcgaaaataaagttagtaatac	NcoI	*wza*	
7792	wza-rev	ccgctcgagttaaactttttcagcttttaac	XhoI	*wza*	
8234	wzx-fw_	cataccatgggatttataaaattagaacaatag	NcoI	*wzx*	
8235	wzx-rev	ccgctcgagtcatgtcaaattattttc	XhoI	*wzx*	
8232	wzy-fw	cataccatgggaatcttaagaatactaatttattcg	NcoI	*wzy*	
8233	wzy-rev	ccgctcgagttaatcatatttttttatttttaac	XhoI	*wzy*	
8236	waaL-fw	cataccatgggaaagtggtataaagaaaattgg	NcoI	*waaL*	
8237	waaL-rev	ccgctcgagttaatctgttgtatctgtttc	XhoI	*waaL*	
7771	uge-fw	cataccatgggaaaaatattagttactggagc	NcoI	*uge*	
7772	uge-rev	ccgctcgagttataaaacatttctgtatatc	XhoI	*uge*	
7773	wbtA-fw	cataccatgggacacatcggtaaaatgacaac	NcoI	*wbtA*	
7774	wbtA-rev	ccgctcgagttagtctatcttatttttatc	XhoI	*wbtA*	
8136	waaL-KOt-1.2	caaaatcaaatgttaaaaaaaatttattgttatttcaagtgtttttc		*waaL*	B
8138	waaL-KOt-3.1	gaaaaacacttgaaataacaataaatttttttaacatttgattttg		*tetQ*	C
8139	waaL-KOt-3.2	gatggttttgtttgtcatattttattttgatgacattgatttttgg		*tetQ*	D
8137	waaL-KOt-2.1	ccaaaaatcaatgtcatcaaaataaaatatgacaaacaaaaccatc		*waaL*	E
7801	wzx-KO-1.1	ggctgcagtactaaaaaaagcaatgaattaatg	PstI	*wzx*	A
7802	wzx-KO-1.2	ctatgatgttgcaaataccgatgagcttctgtatatcttataccat		*wzx*	B
7805	wzx-KO-3.1	atggtataagatatacagaagctcatcggtatttgcaacatcatag		*ermF*	C
7806	wzx-KO-3.2	aaaaactattggtactttttctacgaaggatgaaatttttcagg		*ermF*	D
7803	wzx-KO-2.1	cctgaaaaatttcatccttcgtagaaaaagtaccaatagtttt		*wzx*	E

7804	wzx-KO-2.2	ccactagtctcatttattttccttgtattc	SpeI	*wzx*	F
7910	wzy-KO-1.1	ggctgcagtaatgcaataggaattg ataac	PstI	*wzy*	A
7911	wzy-KO-1.2	ctatgatgttgcaaataccgatgagc cgtattataaatcaaattgtta		*wzy*	B
7914	wzy-KO-3.1	taacaatttgatttataatacggctca tcggtatttgcaacatcatag		*ermF*	C
7915	wzy-KO-3.2	gaattatctattagaattttgctacga aggatgaaatttttcagg		*ermF*	D
7912	wzy-KO-2.1	cctgaaaaatttcatccttcgtagca aaattctaatagataattc		*wzy*	E
7913	wzy-KO-2.2	ccactagtttttcttattatgtgttaatct atta	SpeI	*wzy*	F
7810	waaL-KO-1.1	ggctgcagcacaggaaaacaaaa acagat	PstI	*waaL*	A
7811	waaL-KO-1.2-Ery	ctatgatgttgcaaataccgatgagc tttattgttatttcaagtgt		*waaL*	B
7814	waaL-KO-3.1-Ery	acacttgaaataacaataaagctca tcggtatttgcaacatcatag		*ermF*	C
7815	waaL-KO-3.2-Ery	gatggttttgtttgtcatattctacgaa ggatgaaatttttcagg		*ermF*	D
7812	waaL-KO-2.1-Ery	cctgaaaaatttcatccttcgtagaat atgacaaacaaaaccatc		*waaL*	E
7813	waaL-KO-2.2	ccactagttctattttttccaattgttctg g	SpeI	*waaL*	F
8243	23210-fw	cataccatgggaaaaaaagtacca atagtttttatatttaacc	NcoI		
8244	23210-rev	ccgctcgagtcattttttatcttttttaat atattccac	XhoI		
8245	23290-fw	cataccatgggaattaacaaaattct aatag	NcoI		
8246	23290-rev	ccgctcgagttattttttattttcattag	XhoI		

Table S3. Orthologs of *C. canimorsus* 5 genes encoded in the LOS/CPS loci of Cc2, Cc11 and Cc12 strains

Gene annotation	ORF in Cc5 Ccan_	ORF in Cc2 CCAN2_	ORF in Cc11 CCAN11_	ORF in Cc12 CCAN12_
ugd	23180	1920009	2010004<br>2010005	760037
uge	23190	1920008	2010006<br>2010007	
wzx	23200	1920007<br>1920006<br>1920005	2010008<br>2010009	
glycosyl transferase family 8 A4 GalT_like	23210	1920004		
wfdP	23220	1920003<br>1920002	2010010	
wfdQ	23230	1920001<br>1430001	2010011<br>2010012	
wfdR	23240	1430002	2010013	
glycosyl transferase family 2	23250	1430003	2010014	
glycosyltrans ferase family 1	23260	1430004	2010015	
glycosyltrans ferase family 1	23270		2010016<br>2010017<br>2010018	
wzy	23280		2010019	
glycosyltrans ferase family 1	23290	1430008	2010020<br>10027	
fnlA	23300	1430009	10026	
23S ribosomal RNA	23310	1430010	10025	
wxcM	23320	1430016	10024	
ABC transporter	23330	1840007	2360008	
hypothetycal protein	23340	1840008		
fnlB	23350	1430017	10023	
fnlC	23360	1430018	10022	

wbuB	23370	1430019<br>1430020	10021	
wbtC	23380	1430021	10020<br>10019	
wbtB	23390	1430022	10018	760045
wbtA	23400	10001	10017	760057
rmlA	23410	10002	10016	760058
ATPase	23420	10003	10015	
rmlC	23430	10004	10014<br>10013	760060
rmlD	23440	10005	10012	760061
transposase	23450		10011	
wzz	15540	1850037<br>1850038	2370012	790017
wza	15550	1850039	2370011<br>2370010<br>2370009<br>2370008	790016
